# Psychosis among "healthy" siblings of schizophrenia patients

**DOI:** 10.1186/1471-244X-6-6

**Published:** 2006-01-31

**Authors:** Ritva Arajärvi, Jonna Ukkola, Jari Haukka, Jaana Suvisaari, Jukka Hintikka, Timo Partonen, Jouko Lönnqvist

**Affiliations:** 1 Department of Mental Health and Alcohol Research, National Public Health Institute, Helsinki, Finland; 2 Department of Psychiatry, Helsinki University Hospital, Helsinki, Finland; 3 Päijät-Häme Central Hospital, Lahti, Finland and University of Tampere, Tampere, Finland

## Abstract

**Background:**

Schizophrenia aggregates in families and accurate diagnoses are essential for genetic studies of schizophrenia. In this study, we investigated whether siblings of patients with schizophrenia can be identified as free of any psychotic disorder using only register information. We also analyzed the emergence of psychotic disorders among siblings of patients with schizophrenia during seven to eleven years of follow-up.

**Methods:**

A genetically homogenous population isolate in north-eastern Finland having 365 families with 446 patients with a diagnosis of schizophrenia was initially identified in 1991 using four nationwide registers. Between 1998 and 2002, 124 patients and 183 siblings in 110 families were contacted and interviewed using SCID-I, SCID-II and SANS. We also compared the frequency of mental disorders between siblings and a random population comparison group sample.

**Results:**

Thirty (16%) siblings received a diagnosis of psychotic disorder in the interview. 14 siblings had had psychotic symptoms already before 1991, while 16 developed psychotic symptoms during the follow-up. Over half of the siblings (n = 99, 54%) had a lifetime diagnosis of any mental disorder in the interview.

**Conclusion:**

Register information cannot be used to exclude psychotic disorders among siblings of patients with schizophrenia. The high rate of emergence of new psychotic disorders among initially healthy siblings should be taken into account in genetic analysis.

## Background

Family studies of schizophrenia have established that schizophrenia strongly aggregates in families with a relative risk of about 11 compared with matched comparison groups. When we investigate affected pedigrees, there is a relatively high risk that healthy pedigree members develop schizophrenia or other psychotic disorder in the future, although recent genetic studies of schizophrenia have not investigated this. Further, the current evidence suggests that familial liability to schizophrenia increases not only the risk for schizophrenia as narrowly defined, but also for personality disorders of the schizophrenia spectrum and probably several psychotic illnesses [[1-3], see reviews [4,5]].

Genetic studies of schizophrenia are expensive, mainly because of the need to conduct structured clinical interviews of all family members. If psychotic disorders could be reliably excluded using data from nationwide health care registers, the costs would be greatly reduced as structured interviews would not be needed for all healthy family members.

The aims of this study were to investigate whether siblings of patients with schizophrenia can be identified as not having any psychotic disorder using health care register information only, to analyze the emergence of psychotic disorders among siblings of patients with schizophrenia during seven to 11 years of follow-up, and to compare the prevalence of psychiatric disorders between siblings of patients with schizophrenia and a random population sample.

## Methods

This study was based on a genetically homogenous population isolate in north-eastern Finland where the prevalence and lifetime morbid risk of schizophrenia are substantially higher than elsewhere in the country [6,7], and where chromosomal regions suggesting putative loci for genes predisposing to schizophrenia in families have also been found [8-13].

The study was initially started in 1991, and from the beginning we have collaborated with the Department of Molecular Medicine, National Public Health Institute, Helsinki, Finland. All phases of the study were approved by the review board of Finland's National Public Health Institute, and the Ministry of Social Affairs and Health. All patients and siblings gave informed consent and patients gave permission to contact their family members.

### Registers

In 1991 we used three registers to identify schizophrenia patients born between 1940 and 1969 who had at least one parent born in the internal isolate. The Finnish Hospital Discharge Register records diagnoses and admission and discharge dates for all inpatient stays in both public and private hospitals. The Pension Register records disability pensions and the Free Medicine Register records state subsidized outpatient medication. The registers were computerized in 1969. The registers used the ICD-8 [14] criteria before 1987, the Finnish version of the ICD-9 [15] applying DSM-III-R diagnostic criteria for mental disorders [16] from 1987 to 1995, and the ICD-10 [17] diagnostic codes from 1996. In our own diagnostic reassessment, 69% of the subjects with a register diagnosis of schizophrenia, schizoaffective, or schizophreniform disorder received a diagnosis of schizophrenia, and 87% received any schizophrenia spectrum diagnosis [6]. This accords with another validation study from the isolate sample [18], while in the North Finland 1966 Birth Cohort there have been no false positive schizophrenia diagnosis in the Hospital Discharge Register [19,20].

### Identification of patients and "healthy" siblings

**Figure 1:**
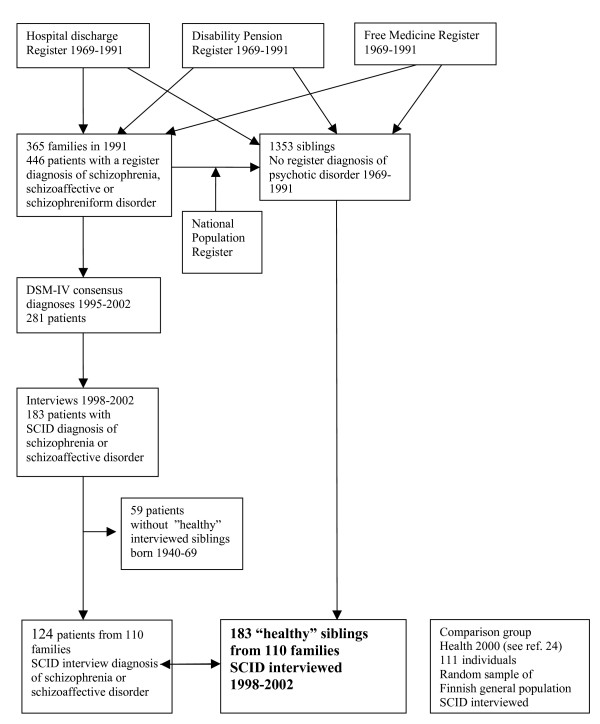
Identification of "healthy" siblings, patients with schizophrenia or schizoaffective disorder, and comparison group.

We identified 365 families with 466 patients with register-based diagnosis of schizophrenia, schizoaffective or schizophreniform disorder and 1353 siblings from the National Population Register (born 1940–1969) with no register-based diagnosis of psychotic disorder (Fig. 1.). Between 1991 and 2002, we contacted these families to collect blood samples for genetic analyses. Of the 446 patients, 69 had deceased, and 82 refused from the blood sample. Personal interviews were not conducted, but DSM-IV [21] diagnoses for affected family members were made using the information in psychiatric case notes from all hospital and outpatient treatment facilities [8,22].

We re-contacted these families between 1998 and 2002 and asked them to participate in face-to-face assessments including an extensive neuropsychological examination and a structured interview. The aim of the follow-up was to ascertain psychiatric diagnoses and to collect more detailed phenotypic data. We also obtained information from the Finnish Hospital Discharge Register on hospital treatments for mental disorders between 1991 and 1998 for both patients and siblings.

We contacted all patients who had previously participated in genetic studies and had a consensus diagnosis. However, 23 patients refused to take part to the interview and tests. We also interviewed siblings of the contacted patients. We could not contact all "healthy" siblings, but for every patient we aimed to interview and test at least one sibling nearest by age [23]. This age criterion was set because we wanted to minimize age-related differences in neuropsychological performance between siblings.

For this study, we included all patients with a SCID interview diagnosis of schizophrenia or schizoaffective disorder (n = 183, Fig. 1.). 124 patients (97 schizophrenia, 27 schizoaffective disorder) had at least one sibling born from 1940 to 1969 who had no register diagnosis of psychotic disorder in 1991 and who was interviewed. 59 patients had no sibling fulfilling the inclusion criteria. The final sample of 110 families with at least one patients-sibling pair consisted of 124 patients and 183 siblings.

Fifty-nine (54%) of 110 families had one interviewed patient and sibling, 24 families had two siblings, 12 three, one four and one six siblings per one patients. In twelve families we had two patients: five families had one sibling, two had two, three had three, and two families had four interviewed siblings. Finally, in one family there were three patients and four interviewed siblings.

### Comparison group for siblings

Comparison group (n = 111) were a representative sample of the Finnish general population aged 30–79 (Fig. 1). The sample was collected from the Health 2000 Survey, which is based on a nationally representative two-stage cluster sample of 8028 persons aged 30 or over [24]. Of the 161 subjects randomly selected from those who had participated in any of the study phases, 111 (68.9 %) were interviewed during 2002–2004 and were taken as comparison group for this study. Information on hospital treatments for any psychotic disorders for them was obtained from the Hospital Discharge Register from 1974 to 2002.

### The interviews and assessments

We interviewed 183 siblings, 124 patients and 111 individuals from the comparison group with SCID-I (Structured Clinical Interview for DSM-IV axis disorders [25] (Fig 1). We interviewed the siblings and the patients with SCID-II [25] and all patients and 113 siblings with the Scale for the Assessment of Negative Symptoms (SANS) [26].

We used the clinician version of SCID-I (DSM-IV), which contains an overview and six modules: mood episodes, psychotic symptoms, psychotic disorders, mood disorders, substance use disorders and anxiety and other disorders. The SCID-I and -II scoresheets were translated into Finnish and we added some questions to the overview scoresheet.

The SANS [26] consists of 25 items and is rated on 0 – 5 scale. It has 5 subscales: affective flattening or blunting, alogia, apathy, asociality, and inattention.

The four teams of field workers had received extensive training in use of the instruments. The reliability for SCID-III-R and SCID-IV has been high [27,28]. To increase reliability we tape-recorded and reviewed the 20 first interviews of each fieldworker. The SCID-I-II and SANS interviews were also reviewed by the senior researcher. The interviews were made on a lifetime basis and blind to the register diagnosis. Comparison group was interviewed using SCID-I only.

### Statistical methods

We calculated the percentages of each mental disorder in the study groups according to the SCID interview data with SPSS 11.0 for Windows. We also counted the frequencies of co-morbid mental disorders, as well as sociodemographic and clinical characteristics like sex, age, education years, current and lifetime psychiatric treatment, and medication.

We took the within-family correlation into account by using conditional logistic regression and general estimation equations (GEE, version 4.13) [29] in our analysis. We had family as the stratification variable, psychosis as the dependent variable and age (40–49, 50–59, 60–69 years), sex, residence (city, population centres, rural), contact for mental health problems, contact for alcohol or substance use problems and smoking as explanatory variables. We also tested the results of 113 SANS interviews of "healthy" siblings using the same logistic regression analysis, with the diagnosis of psychosis as the dependent variable and the SANS items without global ratings as explanatory variables.

We also checked the register diagnoses of 183 interviewed siblings seven years later (31 Dec 1998) to find out if the "healthy" siblings had received any register diagnosis of psychotic disorder in the meantime.

## Results

### Psychotic disorders among siblings of patients with schizophrenia

In the SCID interview there were 30 (16.4 %) siblings with a diagnosis of psychotic disorder among the 183 siblings with no register diagnosis of psychotic disorder in registers in 1991. Diagnosis were one schizophrenia, six schizoaffective psychoses, three delusional disorders, eight psychotic disorders not otherwise specified, and six were psychotic mood disorders (two bipolar and four psychotic major depressive disorders), and six were substance-induced psychotic disorders (four alcohol-induced and two other substance-induced psychotic disorders). Fourteen siblings had had psychotic symptoms before 1991(see Table 1.).

**Table 1:** SCID-I-II principal diagnosis of 183 healthy siblings, and SCID-I diagnosis of 111 individuals from the comparison group. In brackets number of those 14 siblings who had psychotic symptoms before 1991.

	Isolate siblings	Comparison group
N	183	111
**Psychotic disorders**		
Schizophrenia	1 (1) 0.5%	1 0.9%
Schizoaffective disorder	6 (2) 3.3%	
Delusional disorder	3 (1) 1.6%	
Psychotic disorder NOS	8 (4) 4.4%	
Alcohol-induced psychotic disorders	4 (3) 2.2%	1 0.9%
Other substance-induced psychotic disorders	2 (1) 1.1%	
Major depressive disorder with psychotic features	4 (1) 2.2%	
Bipolar disorder I	2 (1) 1.1%	
		
**Other mental disorders**		
Other depressive disorders	28 15.3%	21 18.9%
Personality disorders A *	7 3.8%	not assessed
Personality disorders B-C*	4 2.2%	not assessed
Alcohol use disorders	7 3.8%	14 12.6%
Anxiety disorders	22 12.0%	8 7.2%
Other	1 0.5%	
Diagnosis	99 54.1%	45 40.5%
No diagnosis	84 45.9%	66 59.5%

Total	183 99.9%	111 100%

*5 siblings had SCID-II personality disorder without other diagnosis.

Siblings had 43 (23.5%) co-morbid diagnoses: 15 (8.2%) alcohol use, 15 anxiety (8.2%), and 6 (3.3%) other depressive disorders, 5 (2.7%) personality disorders (2 cluster A and 3 cluster B-C), one anxiolytic dependence disorder and one hypochondriasis. Comparison group had 10 (9.0%) co-morbid diagnoses: 5 anxiety and 5 alcohol use disorders.

The mean age of onset of psychotic symptoms among 30 siblings according to SCID interview was 33.5, SD 8.7 years. Those 14 siblings who had psychotic symptoms before 1991 had the mean age of onset 26.1, SD 9.4 versus 38.4, SD 5.2 years among those 16 siblings with onset of psychotic symptoms in the follow-up. The age at onset distribution ranged from 52 years to one sibling who had had psychotic symptoms already in childhood but could not remember the age of onset.

Siblings who had contacted health care professionals for mental health problems (OR = 7.0, CI 2.6–18.9), for alcohol or substance use problems (OR = 8.8, CI 1.6–49.2) or smoked (OR = 2.4, CI 0.85–6.9) had the highest odds ratios explaining the diagnosis of psychotic disorder. Sex, age, or education years did not predict psychosis among the 183 siblings.

Of all 30 siblings with an interview-based diagnosis of psychotic disorder, seven (23%) had had a hospital treatment because of psychotic disorder between 1991 and 1998 according to the Hospital Discharge Register. However, only one of them belonged to the group that had developed psychotic symptoms before 1991. Less than half (43%) had current psychiatric treatment or medication (Table 2).

**Table 2:** Demographic and clinical characteristics of subjects

	Patients	Psychotic siblings	Non psychotic siblings	Comparison group
N	124	30	153	111
Age at evaluation, y, mean (SD), (min, max)	46.0 (7.3) (31.2 – 64.9)	44.4 (6.3) (32.0 – 61.0)	46.4 (8.8) (24.0 – 72.0)	50.3 (12.3) (30.3–78.0)
Sex of subjects, % male	81 65.3 %	16 53.3%	73 47.7%	56 50.5%
Education, y, mean (SD)	9.5 (2.3)	10.5 (2.1)	11.4 (3.0)	11.7 (4.0)
Ever had psychiatric treatment/medication	123 99.2 %	22 73.3 %	46 30.1 %	31 27.9%
Ever treated in psychiatric hospital	121 97.5%	9 30.0%	11 7.2%	4 3.6 %
Current psychiatric treatment/medication	117 94.4 %	13 43.3 %	19 12.4 %	16 14.4%
Current use of psychiatric medicine	117 94.4 %	13 43.3 %	19 12.4 %	12 10.8%
Antipsychotics	116 93.5 %	8 26.8 %	4 2.6 %	3 2.7%
Antidepressants	25 20.2 %	9 30.0 %	6 3.9 %	4 6.6%
Anxiolytics or sedatives	54 43.5 %	10 33.3 %	8 5.2 %	5 4.5%
GAF current (SD)	36.3 (11.3)	54.9 (11.8)	77.5 (12.5)	79.2 (12.9)

We also analysed the SANS ratings of 113 siblings with logistic regression analysis. The items 3 (paucity of expressive gestures), 7 (lack of vocal inflections), 15 (impersistence at work or school) and 18 (recreational interests and activities) had statically significant odds ratios of 4.6 (CI 0.9–21.8), 2.4 (CI 0.7–8.8), 3.0 (CI 1.8–4.9) and 2.1 (CI 0.9–4.9) respectively, and were associated with the diagnosis of psychotic disorder.

### Non-psychotic disorders among siblings of patients with schizophrenia

There were 69 non-psychotic disorders among siblings of patients with schizophrenia (28 non psychotic depressive, seven personality A, four other personality, seven alcohol use, 22 anxiety disorders, and one sleep disorder, Table 1). In addition, 43 of 183 siblings had co-morbid diagnoses in the SCID interview.

During the follow up, from 1991 to 1998, 11 (7.2%) of interviewed 153 non-psychotic siblings had been hospitalised (Table 2).

### Siblings of patients with schizophrenia compared to comparison group

Demographic and clinical characteristics of siblings (n = 183) and comparison group (n = 111) are presented in Table 2. Individuals in the comparison group were older and had higher current GAF ratings (mean 79.2, SD 12.5 versus mean 73.7, SD 14.9, p = 0.002) than the siblings. Siblings had significantly more diagnoses of psychotic disorders (16% versus 2%, p < 0.0001) than comparison groups in the SCID-I interview (Table 1). In addition siblings did not have more organic diseases or medication than comparison groups.

When we made the analysis using only one interviewed sibling nearest in age to the patients, the result was the same: 14.5% psychoses and 52% mental disorders among siblings.

## Discussion

Our results clearly show that nationwide health care registers cannot be used to exclude psychotic disorders in relatives of patients with schizophrenia. In 1991, when the study was initially launched, 7.7% of the siblings who were presumed to be healthy based on Hospital Discharge Register actually had psychotic symptoms. The number of false negatives would be high enough to jeopardize the results of genetic analyses, if these siblings were treated as unaffected in the analyses. Even if the "healthy" siblings were treated as unknown, as is the current practice, the analyses would have compromised power compared to more exact diagnoses.

The rate of emergence of new psychotic disorders among siblings during the relatively short follow-up time was also high. Between 1991 and 1998, 8.7% of the siblings developed any psychotic disorder, although their mean age in 1991 was already 37 years. The high rate of new-onset psychotic disorders reflects the fact that many siblings came from multiply affected families.

In 1991, many families already had several members with schizophrenia or schizoaffective disorder. The majority of "healthy" siblings had at that time already passed the greatest risk period for schizophrenia, which explains why only one "healthy" sibling received a diagnosis of schizophrenia during the follow-up. However, there were six with schizoaffective disorder and another six with psychotic affective disorders. In two families the schizoaffective and affective psychotic disorders were clustered with three and four affected siblings in the family. We also found three families with two new cases of psychotic disorders. Our findings accord with several family studies which have reported a high risk not only for schizophrenia and schizoaffective disorders but also for affective disorders within families [1-3,30]. Similar findings were seen in a study of extended pedigrees where all patients had a diagnosis of schizophrenia [31].

Although only one sibling received a diagnosis of schizophrenia, particularly the eight siblings with psychosis not otherwise specified might have been presenting premorbid symptoms of schizophrenia, the possibility of which can only be ruled out by a longer follow-up study.

In seven years of follow-up only seven siblings were hospitalized with a diagnosis of psychotic disorder, although all 30 siblings with an interview diagnosis of psychotic disorder had had psychotic symptoms before 1998. So, using the SCID interview allowed us to identify better and earlier the siblings with psychotic disorders. On the other hand the amount of psychiatric inpatient beds decreased dramatically in Finland in 1990s, and this might have lead to the more stringent indications of hospitalization. However, siblings with psychotic disorders had had more contacts to health care professionals for mental health problems and alcohol or substance use problems. This suggest that when persons who have several relatives with schizophrenia or other psychotic disorders seek help for any mental, alcohol or substance use problems psychotic symptoms should always be assessed carefully.

16% of the siblings had a diagnosis of psychotic disorder compared with 2% of the comparison group. Axis-I psychiatric morbidity was common both in siblings (51%) and in comparison groups (41%). The frequency of anxiety disorders was higher among siblings than comparison group, contrary to some other studies [5,31]. In addition, siblings more often had co-morbid diagnoses (24%) than the comparison group (6%), suggesting that their clinical status was more complicated and they were possible more seriously affected.

"Paucity of expressive gestures" and "lack of vocal inflections" as negative symptoms are phenotypic features that could benefit genetic analysis in addition to clinical diagnosis. We found in our previous studies that patients with first-degree relatives with psychotic disorder had more severe negative symptoms than patients without familial loading for schizophrenia [32].

### Limitations

Research environment in Finland is unique because of the extensive health care registers, which allow us to identify and follow up patients. However, findings based to register data are not generalizable to countries without such registers.

Comparison group is a representative sample of the Finnish general population but it might not be representative of the population originating from the isolate. We measured only DSM-IV axis I, but did not use SCID-II.

We did not calculate the reliability of the interview method, but in our earlier study the SCID interview identified more affective disorders than the best estimate consensus diagnoses [6]. However, our interviewers were well-trained and they reviewed the interviews with senior researchers. Patients were interviewed before the siblings, and siblings and interviewers were not aware of patients' diagnoses. Nevertheless, it was difficult for the interviewers to remain completely blind to the status of family members, because the participants often wanted to discuss about the situation in their family.

Negative symptoms were measured with SANS, but only 113 of 183 siblings and no comparison groups were interviewed. Moreover, SANS has not been validated or even used in general population. The reliability of SANS has been evaluated by Andreasen and colleagues as excellent [33,34]. However, Norman and colleagues [35] found that the interrater reliability on the whole was lower than reported earlier. So our result that paucity of expressive gestures associate with psychosis in siblings is preliminary.

## Conclusion

Register information cannot be used to exclude psychotic disorders among siblings of patients with schizophrenia. Furthermore, during a relatively short follow-up, 8.7% of initially healthy siblings developed some psychotic disorder. The high rate of emergence of new psychotic disorders among initially healthy siblings should be taken into account in genetic analysis, and it also encourages the use of endophenotypes instead of reliance on psychiatric diagnoses alone.

## Competing interests

The author(s) declare that they have no competing interests.

## Authors' contributions

The authors wish to thank all the patients and their families who participated in this study. All field workers are thanked for contacting the patients and their families for the interviews.

## Pre-publication history

The pre-publication history for this paper can be accessed here:


